# Editorial: Exploring the psychological therapies: prevention and intervention for suicidal attempt, ideation, behavior in adolescents

**DOI:** 10.3389/fpsyt.2026.1798547

**Published:** 2026-02-05

**Authors:** Sami Hamdan, Wenchao Wang, Xiaobo Xu

**Affiliations:** 1 Academic College Tel Aviv-Jaffa, Tel Aviv Jaffa, Israel; 2 Beijing Normal University, Beijing, China; 3 Shanghai Normal University, Shanghai, China

**Keywords:** adolescents, depression, emotion regulation, mental health interventions, non-suicidal self-injury (NSS), risk factors, suicidal ideation (SI)

The rising prevalence of suicidality and self-injurious behaviors among adolescents and young adults remains an urgent challenge for global mental health systems. Across cultures and clinical contexts, this vulnerable population faces complex interactions among psychological, social, and environmental risk factors. In this Research Topic, we bring together a collection of studies that reflect the diversity of perspectives, methodological approaches, and intervention strategies in this field. Collectively, these contributions deepen our understanding of self-injury and suicidal behavior while offering practical insights for prevention and care.

A recurring theme across the articles is the central role of non-suicidal self-injury (NSSI) as both an independent concern and a potential precursor to suicide. In their study of medical students, Lin et al. examined how social support buffers the impact of negative life events on NSSI. Interestingly, they found that urban students were more vulnerable, exhibiting a more pronounced decline in social support following life stressors than their rural peers, challenging conventional assumptions about urban resource advantages.

Gender emerged as another crucial moderator in several studies. Fang et al. revealed that obsessive-compulsive symptoms predict suicide risk in adolescents, mediated by depressive symptoms across genders, with sleep disturbances serving as an additional mediator only in females. Their findings emphasize the need for gender-sensitive assessments in adolescent psychiatric care.

From a developmental perspective, the work by Goldstein et al. introduced valuable age-based nuance through the lens of the Interpersonal Theory of Suicide. Among females with eating disorders, they found that perceived burdensomeness was a stronger predictor of suicidality in adolescents, whereas thwarted belongingness was more influential among emerging adults. These findings support age-specific interventions, such as enhancing social integration among young adults and focusing on family dynamics in adolescents.

The ecological context of youth mental health also received significant attention. Pastor et al. found that, in a structural equation model, family connectedness was the only significant predictor of reduced depression and suicide risk among Spanish adolescents. School and peer connectedness, though relevant, had less predictive power. These findings challenge the growing trend toward school-based prevention models and redirect attention to the family unit as the primary site for intervention.

At the systems level, Parpio et al. conducted a comprehensive scoping review of school-based suicide-prevention programs. Their synthesis highlights the effectiveness of integrated, multi-tiered approaches that combine student education, digital interventions, teacher training, and family-based support. However, they also highlight the scarcity of culturally adapted interventions in low- and middle-income countries, emphasizing the need for future efforts to focus on contextual relevance and accessibility.

This geographic gap is further illustrated in the meta-analysis by Bezie et al., who focused on African students. They found that students exposed to bullying were 1.7 times more likely to report suicidal ideation. Their work highlights the wider socio-cultural and structural factors contributing to youth suicidality in under-resourced schools and advocates for comprehensive anti-bullying policies and psychosocial support programs across African educational institutions.

Yuan et al. used a longitudinal design to examine how mindfulness and emotion regulation strategies influence NSSI and suicidal ideation in Chinese adolescents. They found that cognitive reappraisal mediated the effect of mindfulness on suicidal ideation, whereas both reappraisal and suppression mediated the link to NSSI. This suggests that mindfulness-based interventions could be a valuable approach for early prevention efforts focused on adolescents’ emotional well-being development.

Finally, Yin et al. analyzed how peer victimization relates to NSSI and found that gender plays a crucial role in these links. Social and verbal victimization were linked to NSSI in males, whereas physical and property-related victimization had a greater influence on females. Their results advocate for the adoption of gender-sensitive, trauma-informed strategies in both clinical and educational environments.

Taken together, the studies in this Research Topic confirm that suicidal ideation and self-injury in young people are influenced by multiple factors: developmental stage, gender, sociocultural context, family and school support, psychopathological symptoms, and emotion regulation skills. As a result, interventions need to be comprehensive, adaptable, context-specific, and tailored to individual needs.

We are grateful to all contributing authors for their valuable research and acknowledge the peer reviewers for their constructive feedback in enhancing the work. As Topic Editors, we aim for this Research Topic to inspire future research and to guide interventions that are both compassionate and grounded in evidence.

